# Supplementary material for: Jasmonate primes plant responses to extracellular ATP
Source: Plant J. 2025 Oct 6;124(1):e70514. doi: 10.1111/tpj.70514 (PMC12498120; doi:10.1111/tpj.70514)
Supplement: Supplementary file 1 — Figure S1. Exogenous ATP treatment does not reduce JAZ1 protein stability in aos mutant seedlings, while MeJA does. Figure S2. The time to maximal ATP‐induced [Ca2+]cyt response is reduced after MeJA treatment. Figure S3. Effect of overnight DMSO treatment on ATP‐induced changes in [Ca2+]cyt. Figure S4. Effect of water, DMSO, or MeJA on [Ca2+]cyt. Figure S5. Effect of NaCl or MeJA on ATP release from leaf discs. Figure S6. Induction of P2K1 by JA may be mediated by MYC transcription factors. Figure S7. JA‐primed eATP‐responsive gene expression. Table S1. Reduced induction of P2K1 gene by MeJA treatment in the myc2 mutant. Table S2. Primers used in this study. [file TPJ-124-0-s001.docx]

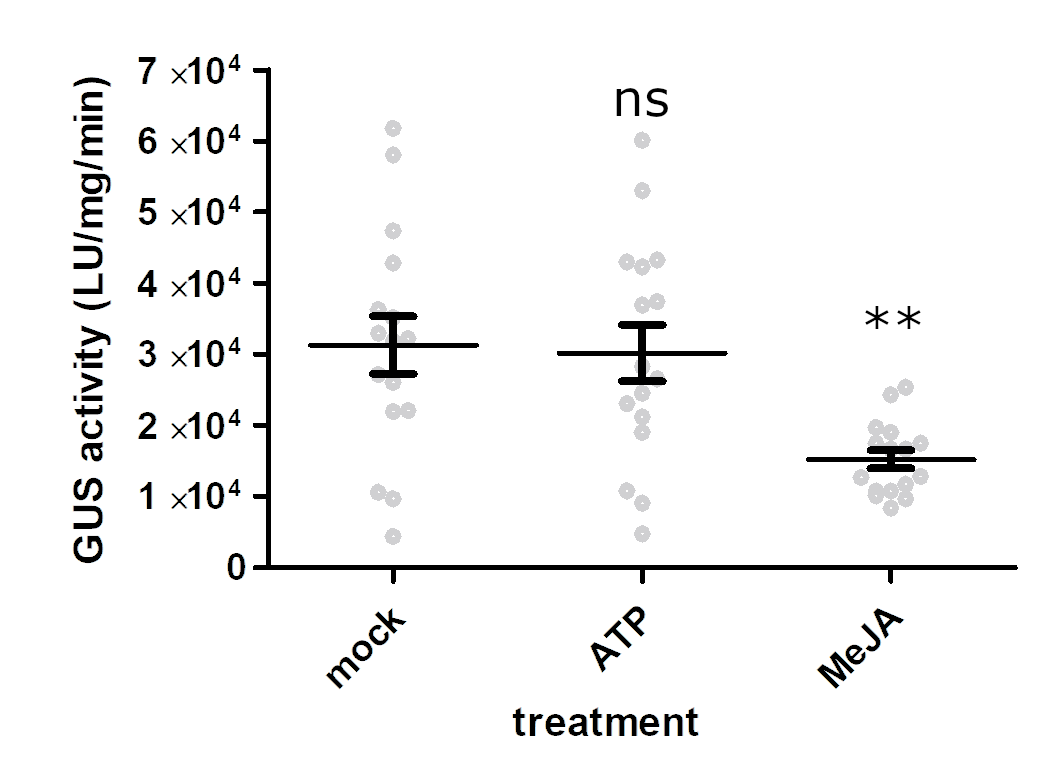


**Supplemental Figure S1. Exogenous ATP treatment does not reduce JAZ1 protein stability in *aos* mutant seedlings, while MeJA does.** Mutant *aos* seedlings expressing JAZ1-GUS were treated with 2 µM MeJA or 1 mM ATP for 30 minutes and processed for quantitative GUS activity measurements to assess JAZ1 protein stability as described in Methods. Statistical comparisons to mock-treated seedlings are indicated by ** (*P <* 0.01) and ns (*P* = 0.97, Fisher’s LSD). Error bars indicate SEM, *n*=16 replicates per treatment across 2 trials.


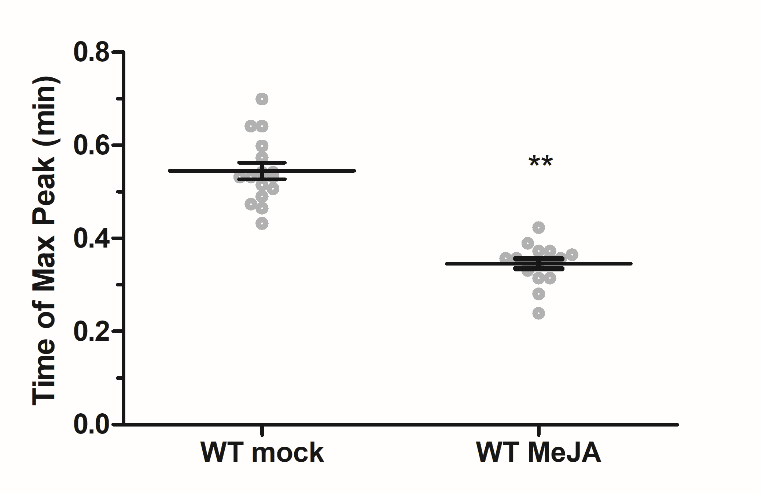


**Supplemental Figure S2. The time to maximal ATP-induced [Ca^2+^]_cyt_ response is reduced after MeJA treatment.** Calcium values from Figure 2B were manually inspected for the time of maximum calcium level. MeJA pretreatment resulted in ~37% earlier maximal calcium response in WT seedlings (*** *P <* 0.001, two-sided t-test). Error bars indicate SEM, *n*=16.

**
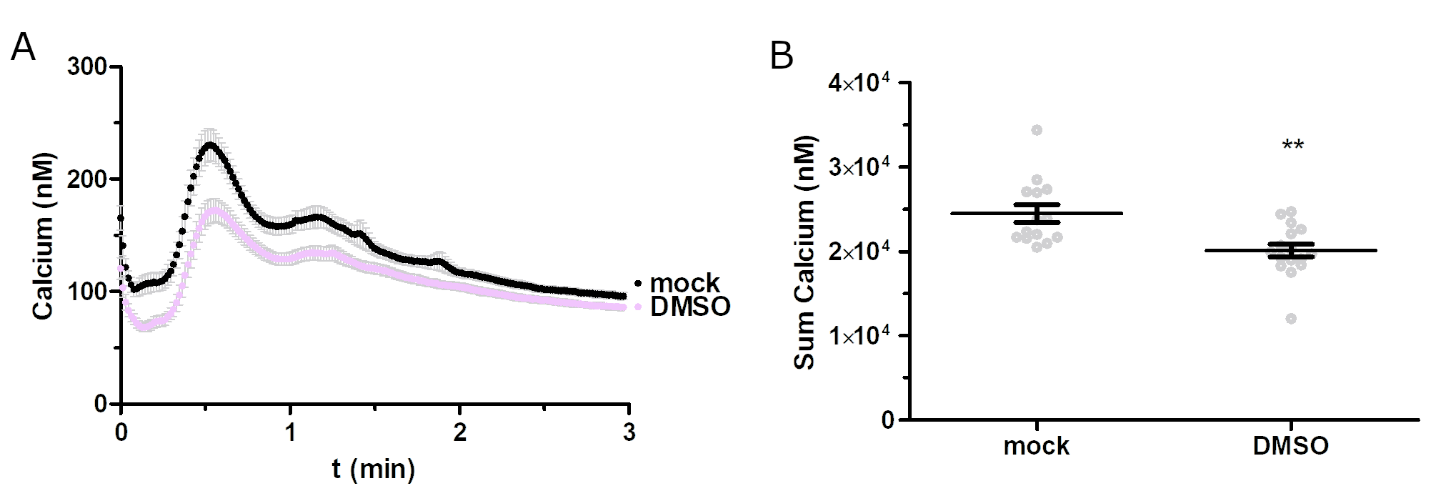
**

**Supplemental Figure S3. Effect of overnight DMSO treatment on ATP-induced changes in [Ca^2+^]_cyt_. (A)** Seedlings were incubated in reconstitution buffer with or without 0.05% DMSO overnight and treated with 100 µM ATP and aequorin luminescence was recorded for 3 minutes. **(B)** Summed calcium values from panel A. DMSO treatment resulted in ~18% reduction in calculated calcium values (** *P* < 0.01, two-sided t-test). Error bars indicate SEM, *n*=14-16 seedlings.

**
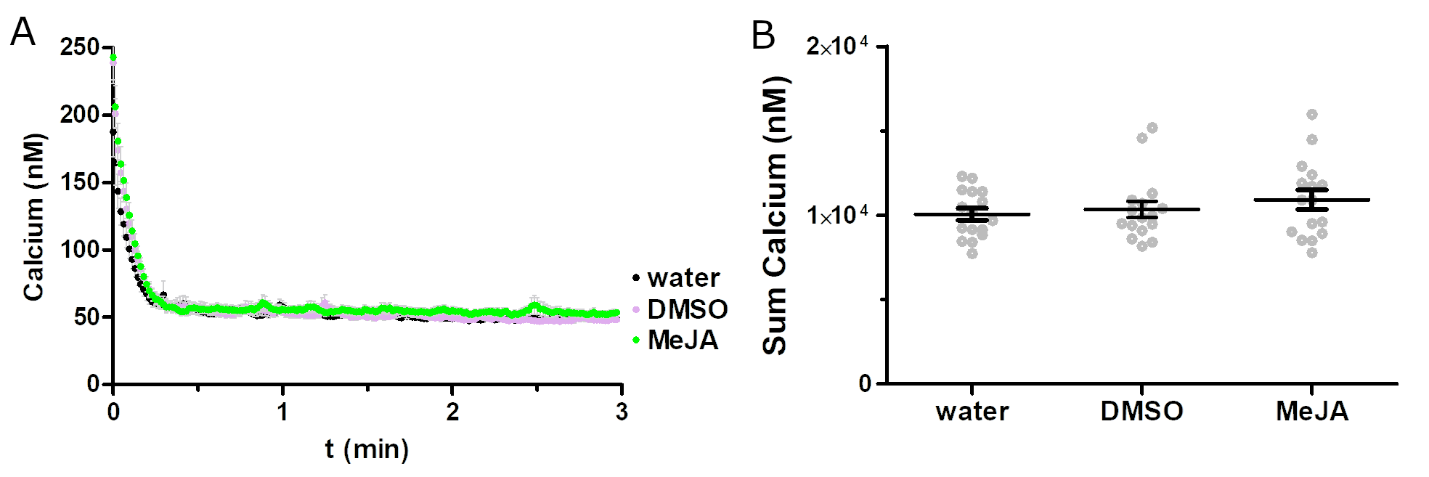
**

**Supplemental Figure S4. Effect of water, DMSO, or MeJA on [Ca^2+^]_cyt_. (A)** Seedlings were incubated overnight in reconstitution buffer then treated with water, 0.05% DMSO, or 50 µM MeJA and aequorin luminescence was recorded for 3 minutes. **(B)** Summed values from panel A. There were no significant differences in total calcium levels between the treatments (*P* > 0.4, one factor ANOVA). Error bars indicate SEM, *n*=16 seedlings.

**
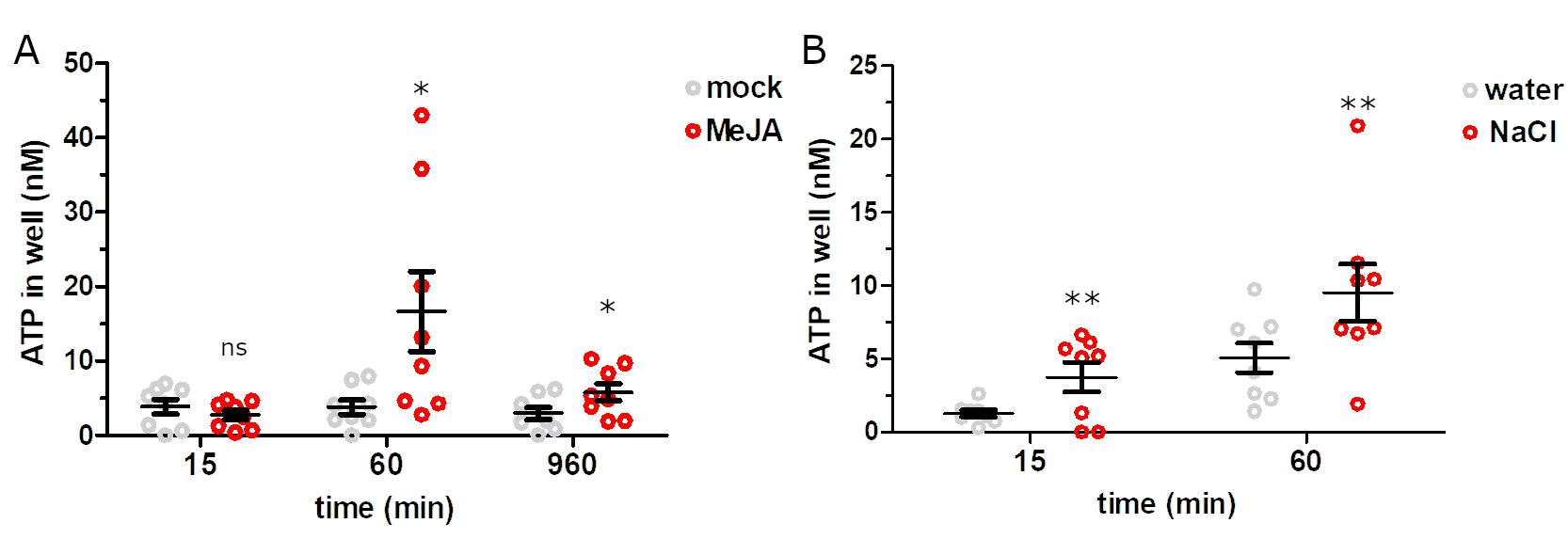
**

**Supplemental Figure S5. Effect of NaCl or MeJA on ATP release from leaf discs.** Leaf discs were treated at time 0 with **(A)** water or 200 mM NaCl, or **(B)** 0.05% DMSO or 50 µM MeJA and incubation media was collected at the indicated time points and assayed for ATP concentration. Error bars indicate SEM, *n*=8 leaf discs. (ns *P >* 0.1, * *P <* 0.05, ** *P* < 0.01, 1-sided t-test.)


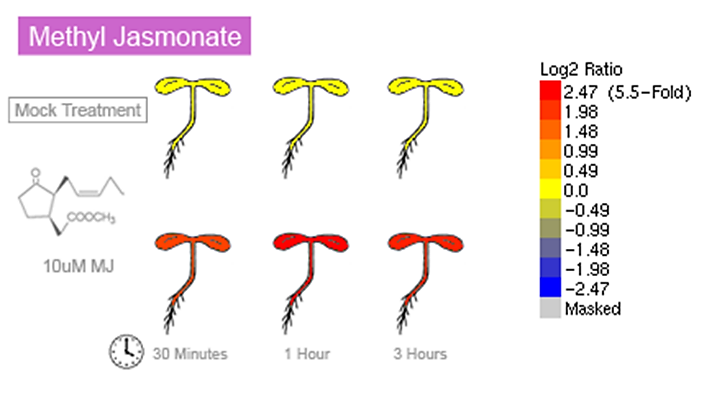

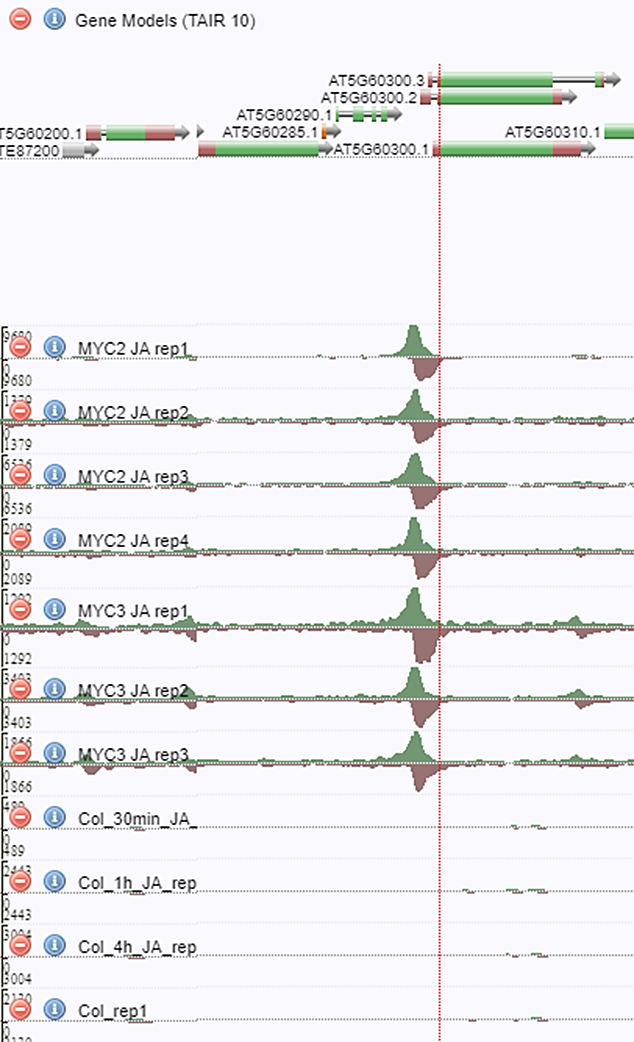


**A**

**B**

**Supplemental Figure S6. Induction of P2K1 by JA may be mediated by MYC transcription factors. (A)** Data from the Arabidopsis eFP Browser (Winter et al., 2007) shows strong *P2K1* upregulation by MeJA treatment, visualized at <http://bar.utoronto.ca/efp/cgi-bin/efpWeb.cgi>. **(B)** ChIP-seq data (Zander et al., 2020) demonstrate specific MYC2 and MYC3 binding to the promoter region of *P2K1* (AT5G60300), visualized at <http://neomorph.salk.edu/MYC2>. Note that the Y-axis for MYC data is on the order of thousands of reads, while the Col-0 data is on the order of 100s.


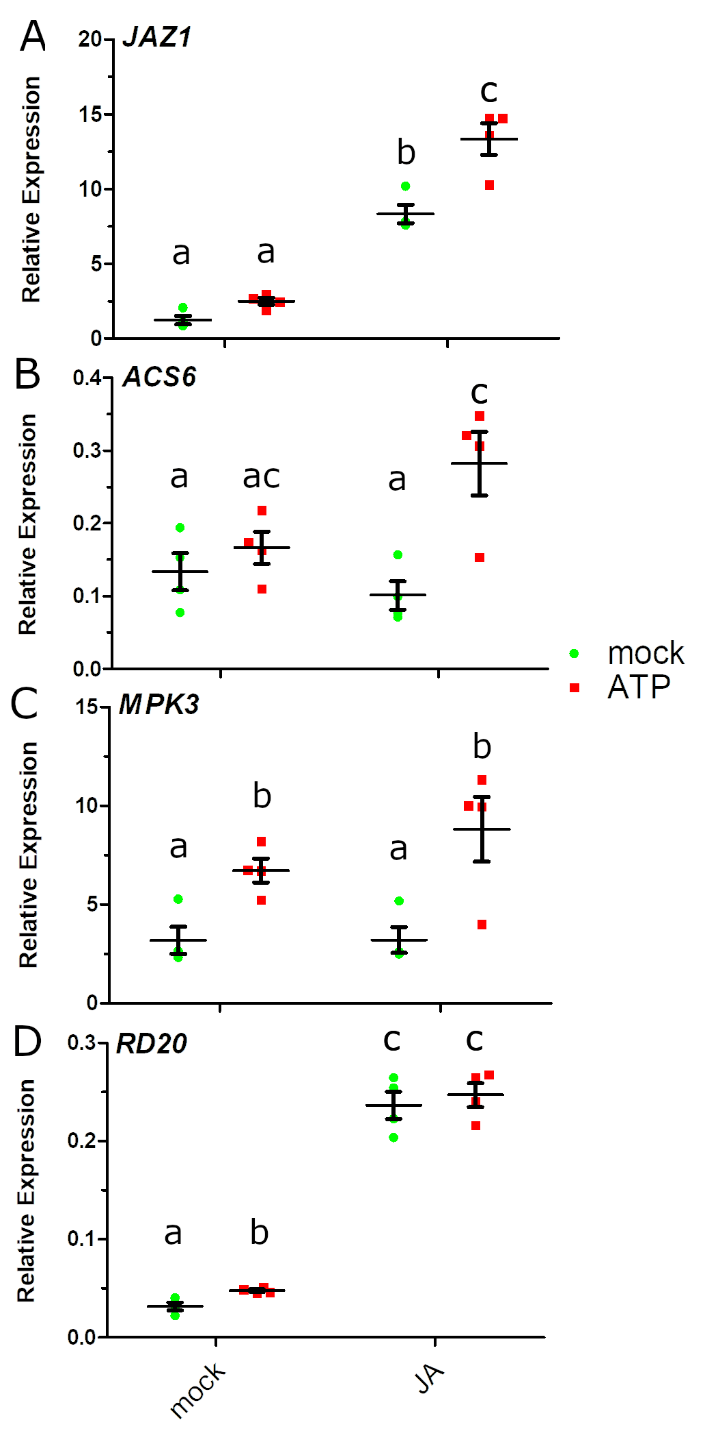


**Supplemental Figure S7. JA-primed eATP-responsive gene expression.** Wild-type seedlings were pre-treated with or without 20 µM MeJA, followed by 0.5 mM ATP treatment as described in Materials and Methods. Gene expression was evaluated by RT-qPCR for **(A)** *JAZ1*, **(B)** *ACS6,* **(C)** *RD20*, and **(D)** *MPK3*. Mean ± SEM and individual values are shown with different letters indicating statistically significant differences in gene expression (*n* = 4, *P* < 0.05, Tukey HSD).

**Supplemental Table S1. Reduced induction of *P2K1* gene by MeJA treatment in the *myc2* mutant.** Data from Zander et al., 2020.

| Time after JA treatment (minutes) | Fold change of *P2K1* expression (Col-0/*myc2*) | FDR |
| --- | --- | --- |
| 0 | 1.15 | 0.46 |
| 30 | 1.30 | 0.04 |
| 120 | 1.78 | 1.2e-5 |
| 240 | 0.96 | 0.86 |

**Supplemental Table S2. Primers used in this study.**

| Primer | Sequence 5’-3’ | Reference |
| --- | --- | --- |
| qJAZ1 f | GAGCAAAGGCACCGCTAATA | Grunewald et al. 2009 |
| qJAZ1 r | TGCGATAGTAGCGATGTTGC |  |
| qMPK3 f | GCTTGGCACACCGACAGAATC | Choi et al. 2014 |
| qMPK3 r | CGTGGGAAGTTGGGAAGTTGC |  |
| qP2K1 f | TGGAGTTTGTCAGGTCCATCG | Choi et al. 2014 |
| qP2K1 r | CTGAGGATCTTCTGCAGGCAA |  |
| qPP2A f | TAACGTGGCCAAAATGATGC | Czechowski et al. 2005 |
| qPP2A r | GTTCTCCACAACCGCTTGGT |  |
| qRD20 f | TGACACCGAAGGAAGGTATGTCC | Choi et al. 2014 |
| qRD20 r | CTTTAACCGTTAGCGCGTATTTGC |  |
| qCPK28 f | ACCCACGAGCACGGCTAA | Choi et al. 2014 |
| qCPK28 r | TTCTCTAACCCACGCATGTGAT |  |
| qRBOHD f | CATGCGGGTGCCCATTT | Choi et al. 2014 |
| qRBOHD r | ATCCGCGGCAATTAAACG |  |
| qLOX3 f | GCTCGCTAAAGCCCACGTTAGTTC | Choi et al. 2014 |
| qLOX3 r | AGCATGCATGTGTCCGTAACCAG |  |
| qACS6 f | CAGCAACGTTTGATTCGGAAA | Choi et al. 2014 |
| qACS6 r | CGTTGAGCTTCACTTGGTGAAC |  |

**References Cited in Supporting Information**

Choi J, Tanaka K, Cao Y, Qi Y, Qiu J, Liang Y, Lee SY, Stacey G. (2014) Identification of a plant receptor for extracellular ATP. Science 343:290-294.

Czechowski T, Stitt M, Altmann T, Udvardi MK, Scheible W-R. (2005) Genome-wide identification and testing of superior reference genes for transcript normalization in Arabidopsis. Plant Physiology 139:5-17.

Grunewald W, Vanholme B, Pauwels L, Plovie E, Inzé D, Gheysen G, Goossens A. (2009) Expression of the Arabidopsis jasmonate signalling repressor JAZ1/TIFY10A is stimulated by auxin. EMBO Reports 10:923-928.

Winter D, Vinegar B, Nahal H, Ammar R, Wilson GV, Provart NJ. An “electronic fluorescent pictograph” browser for exploring and analyzing large-scale biological data sets. PloS ONE 2(8):e718
